# Factors affecting the disclosure of diabetes by ethnic minority patients: a qualitative study among Surinamese in the Netherlands

**DOI:** 10.1186/1471-2458-11-399

**Published:** 2011-05-27

**Authors:** Mirjam JE Kohinor, Karien Stronks, Joke A Haafkens

**Affiliations:** 1 Department of Public Health, Academic Medical Centre, Amsterdam, the Netherlands; 2 Department of General Practice, Academic Medical Centre, Amsterdam, the Netherlands

## Abstract

**Background:**

Diabetes and related complications are common among ethnic minority groups. Community-based social support interventions are considered promising for improving diabetes self-management. To access such interventions, patients need to disclose their diabetes to others. Research on the disclosure of diabetes in ethnic minority groups is limited. The aim of our study was to explore why diabetes patients from ethnic minority populations either share or do not share their condition with people in their wider social networks.

**Methods:**

We conducted a qualitative study using semi-structured interviews with 32 Surinamese patients who were being treated for type 2 diabetes by general practitioners in Amsterdam, the Netherlands.

**Results:**

Most patients disclosed their diabetes only to very close family members. The main factor inhibiting disclosure to people outside this group was the Surinamese cultural custom that talking about disease is taboo, as it may lead to shame, gossip, and social disgrace for the patient and their family. Nevertheless, some patients disclosed their diabetes to people outside their close family circles. Factors motivating this decision were mostly related to a need for facilities or support for diabetes self-management.

**Conclusions:**

Cultural customs inhibited Surinamese patients in disclosing their diabetes to people outside their very close family circles. This may influence their readiness to participate in community-based diabetes self-management programmes that involve other groups. What these findings highlight is that public health researchers and initiatives must identify and work with factors that influence the disclosure of diabetes if they are to develop community-based diabetes self-management interventions for ethnic minority populations.

## Background

Type 2 diabetes is an increasingly common chronic condition all across the globe. Many ethnic minority populations living in the United States and Europe exhibit a greater risk of developing diabetes and diabetes-related morbidity and mortality than people of European origin [1-4]. Reducing ethnic disparities in diabetes-related morbidity and mortality has therefore become an important public health issue on both sides of the Atlantic. Careful diabetes self-management (such as monitoring blood glucose, using medication, following a diet, and exercising) is a key factor in preventing diabetes-related complications in all ethnic groups [2,5]. Diabetes self-management education is considered to be the cornerstone of diabetes care [6]. Studies in the US based on ecological approaches to chronic care have shown that - particularly in ethnic minority populations - the outcomes of clinic-based diabetes care and education can be strengthened by complimentary community-based social support interventions [7,8]. For instance, a recent controlled trial among African Americans found that a church-based diabetes self-management programme improved short-term metabolic control, diabetes knowledge, and diabetes-related quality of life [9]. Several other studies demonstrated significant results from community-based diabetes-self care interventions among Latinos, Mexican Americans, and African Americans [10,11]. Given these US data and ongoing outreach initiatives [12-15], public health researchers in Europe are also becoming increasingly interested in community-based interventions as a possible strategy for improving diabetes self-management and diabetes-related health outcomes in ethnic minority populations [16,17].

To participate in community-based interventions, however, patients must be willing to disclose their condition to people in their social networks [18]. Previous research has indicated that patients' decisions to share information about chronic conditions with others are the outcome of a complex process that includes the consideration of many different issues (including the severity of their condition, the visibility of symptoms to others, the fear of stigmatisation, and the potential benefits of support by others) [19-21]. There is also some evidence that young people with diabetes are not always willing to discuss their disease with others [22], and that cultural norms and traditions may present specific barriers to the disclosure of diabetes in some ethnic minority populations [23,24].

According to Netto [17], one of the five principles for planning targeted interventions for minority ethnic communities includes identifying barriers to access to and participation in such interventions. If public health initiatives are to apply community-based interventions as a strategy to improve diabetes self-management in ethnic minority patients [14,16], a thorough understanding is needed of factors that influence patients' decisions to disclose their diabetes to people in their social networks. In Europe, research on interventions that may help reduce ethnic disparities in diabetes outcomes has only recently begun, and the evidence base is still very limited [13]. American studies on the effects of community-based interventions cannot automatically be generalised to ethnic minority populations in Europe.

Surinamese of South Asian and African descent living in the Netherlands are two migrant groups with a high incidence of diabetes and diabetes-related complications and mortality [1,25,26]. A recent Dutch study reported an age-standardised prevalence of type 2 diabetes in Surinamese of African and South Asian descent of 14.2% and 26.7% respectively, compared with 5.5% in Dutch individuals of European descent. Another Dutch study observed an increased relative risk of death from diabetes among the Surinamese population (1.14 (0.90-1.44) versus 5.29 (4.48-6.25)) [25].

To gain further insight into the factors that influence decisions regarding the disclosure of diabetes by ethnic minority patients, our study explored the perceptions of Surinamese patients with type 2 diabetes on sharing information about their diabetes with family members and people in their wider social networks. We focused especially on factors that either inhibit or motivate the disclosure of this information.

## Methods

To elicit information on how patients viewed and managed the disclosure of diabetes to people around them, we undertook a qualitative study based on in-depth individual interviews that were guided by a topic list. Such interviews are particularly useful for exploring patients' own ideas, as they give respondents the opportunity to address themes that researchers may not have anticipated [27,28].

### Participants

Surinamese are the third largest ethnic minority group in the Netherlands. In the years leading up to the independence of Suriname in 1975, nearly one-third of Suriname's population emigrated to the Netherlands. Due to the country's complex migration history, the ethnic composition of the Surinamese population is diverse. The largest ethnic group is of African descent. They descend from West Africans who were taken to Suriname during the slave trade era. The South Asian Surinamese, also known as Hindustani, are the second largest ethnic group and are descendants of indentured labourers brought to Suriname from northern India in the nineteenth century. As a consequence of its colonial history, Dutch is still the official language in Suriname, and is used in education, government, business, and the media.

Patients were recruited from two general practices in south-eastern Amsterdam. In 2009, 33% of the 79,000 residents in this neighbourhood were of Surinamese origin. Our aim was to recruit 30 to 35 patients. This number is generally sufficient to achieve data saturation, which is a criterion for sample size in qualitative studies [29,30]. Patients were eligible if they met the following criteria: between 30 and 70 years of age, a diagnosis of type 2 diabetes, and of South Asian or African Surinamese origin. Within this group, we sought maximum balance with respect to health centre, ethnicity, and sex.

Electronic patient records from the general practices were used to generate a list of all patients who met our inclusion criteria. Because Dutch patient records provide no information on patients' ethnic background, a practice nurse assessed the ethnicity of the selected patients using their names. On the basis of our inclusion criteria, the researcher invited 74 patients for an interview by mail. Two patients declined participation by means of the enclosed reply card. Those who did not decline by mail (n = 72) were telephoned. Of this group, 45 could be reached: 4 patients did not identify themselves as Surinamese, 9 refused, and 32 agreed to participate in the interview. All patients gave verbal informed consent on tape to participate in the study.

### Data collection and analysis

The topic list for the interviews was built on data from a short field exploration and earlier research on patient perspectives on cardiovascular risk factors [31,32]. It consisted of a range of open-ended questions exploring patients' perspectives on diabetes and diabetes management. The data collection and analysis was an iterative process, meaning that results from the analysis of the first interviews were used to adapt the interview guide if necessary to clarify themes. One of the topics addressed in the first interviews was whether respondents had experienced support from family members, friends, or others in the management of their diabetes. Many respondents answered the interviewer that they had not disclosed their diabetes to most of the people they knew. For this reason we decided to explore this issue more extensively in the subsequent interviews. The first author conducted the interviews from April 2008 to August 2008. All but three interviews were held in the respondents' homes. The interviews lasted one hour on average.

Interviews were recorded and transcribed verbatim. Transcripts were analysed with grounded theory methods and MAXQDA software [33]. For this article, we selected and coded excerpts containing the respondents' statements about the disclosure of diabetes to people in their social networks. The coding process began with assigning codes to statements in the selected excerpts from the interviews. To make the analysis more workable, codes or statements referring to common themes were grouped into similar concepts. Broader categories and subcategories were generated from these concepts through a process of constant comparison or verification. Text excerpts referring to reasons for disclosure and reasons for non-disclosure were analysed separately. The final outcome of this coding process was a data-based thematic matrix or framework of categories, subcategories, and codes, which summarised perceived factors for the disclosure or non-disclosure of diabetes.

MK coded the transcripts. To increase validity, the following procedures were used: two of the researchers (MK and JH) independently coded one interview transcript to check for inter-coder consensus. MK and JH independently checked thematic matrixes to check for consensus about the categories and subcategories that emerged from the data. Final analyses and matrixes were then reviewed by KS.

### Ethics

In line with the code of good conduct in medical research of the Academic Medical Centre(AMC), of the University of Amsterdam provisions were made to assure the anonymity of the respondents in the collection, analysis, and presentation of the data [34]. The study protocol was successfully submitted to the AMC's Medical Ethics Committee [35] (reference 10.17.0530).

## Results

We interviewed 32 patients: 16 African Surinamese and 16 South Asian Surinamese. Table 1 presents the characteristics of the respondents. All respondents were first-generation migrants, but most had been living in the Netherlands for a substantial part of their lives (mean 29 years, range 3-41 years). The duration of diabetes varied from 1 to 28 years, with a mean of 9 years. All respondents were currently being treated for diabetes by a general practitioner (GP) and being seen by a nurse practitioner on a regular basis. Almost one-third of the respondents (31%) lived alone, while the others shared their household with a spouse and/or children. The majority (98%) reported being religious, mostly Hindu or Christian.

**Table 1:** Socio-demographic and clinical characteristics of respondents

Characteristics	Participants (n = 32)
Mean age (mean; range) (years)	55 (36-70)

Gender	
Male	12 (38%)
Female	20 (62%)

Educational level:	
None or primary education	7 (22%)
Lower or general vocational education	9 (28%)
Intermediate or higher general education or intermediate vocational training	12 (38%)
Higher vocational college or university	4 (12%)

Ethnicity	
South Asian Surinamese	16 (50%)
African Surinamese	16 (50%)

Household composition	
Living alone	10(31%)
Living with spouse	6(19%)
Living with spouse and children	9 (28%)
Living with children	7 (22%)

Religion	
Hindu	14 (44%)
Christian	11 (34%)
Muslim	2 (6%)
Other	3 (9%)
None	2 (6%)

Duration of residence in the Netherlands (mean; range) (years)	29 (3-41)

Duration of diabetes (mean; range) (years)	9 (1-28)

Current diabetes medication use	
None	
never started	1(3%)
stopped	3(9%)
Oral medication	22(69%)
Insulin	6(19%)

The interviews yielded 225 statements on how the participants thought about disclosing diabetes to those in their social networks. Participants' initial responses to the question about who they had talked to about their diabetes often elicited answers like *I don't like to talk about my diabetes*. From the further analysis, several common categories and subcategories emerged that were perceived as factors for inhibiting or motivating the disclosure of diabetes. Tables 2 and 3 show the categories, subcategories, and corresponding concepts for factors respectively inhibiting and motivating disclosure.

**Table 2:** Inhibitors affecting the disclosure of diabetes: thematic matrix of categories, subcategories, and concepts (n = 32)

Category	Subcategory	Concepts
Perception of social environment	Community conventions	• Don't burden others with your disease. (n = 5)^1^ • Sickness is private, talking about it is taboo. (n = 8)
	Social consequences	• People gossip. (n = 7) • Fear of bringing shame on self/family. (n = 8) • Career concerns. (n = 4)

Perception of diabetes	Severity of condition	• My diabetes is not severe, so I can take care of my diabetes myself. (n = 4)
	Non-acceptance	• I have difficulty accepting I have diabetes. (n = 3)

Perception of diabetes management	Individual responsibility	• Taking care of one's diabetes is primarily an individual responsibility. (n = 4)

^1 ^n refers to the number of participants who mentioned this concept. The total n for a category may be > 32 because respondents could mention more than one concept.

**Table 3:** Motivators affecting the disclosure of diabetes: thematic matrix of categories, subcategories, and concepts (n = 32)

Category	Subcategory	Concepts
Perceptions of social environment	Close family member	• Only close relatives can be trusted because they will keep it to themselves. (n = 9)^1^ • Family gives social support. (n = 6)
	Peers	• Others with diabetes can give advice. (n = 3)

Perceptions of diabetes	Acceptance	• Once I accepted it myself, I was ready to talk about it. (n = 6)

Perceptions of diabetes management	Precautionary measure	• People should know in case something happens. (n = 5)
	Requirements of therapeutic regimes	• I need certain facilities for my diabetes. (n = 3)
	Disclosure improves social support	• People are considerate about my situation. (n = 3)

^1 ^n refers to the number of participants who mentioned this concept. The total n for a category may be >32 because respondents could mention more than one concept.

In the following sections, we will further describe the inhibitors and motivators affecting disclosure

### Perceptions of the social environment

In reflections on who they had talked to about their diabetes, South Asian and African Surinamese respondents often referred to prevailing conventions in the Surinamese community with regard to the disclosure of disease. A 64-year-old African Surinamese man (ID 25) stated: *I don't talk about diabetes very much. If Surinamese people have a problem they keep it private, within closed doors. People don't talk. You aren't obliged to talk. Dutch people are even willing to expose their problems on TV. Surinamese don't do this. Only Surinamese people who've been living in Holland for a very long time ... They might do this. But those people are almost born here and then their mentality changes somewhat*.

A 60-year-old South Asian Surinamese man (ID 31) explained that talking about diseases is seen as taboo in his community: *[South Asians] are like that. They think it's taboo. They wouldn't tell you. Neither do I, I don't dare tell anyone that I'm a diabetic*.

Respondents also mentioned fear of social consequences as a reason for keeping quiet about diabetes to people outside their households. According to a 70-year-old South Asian Surinamese man (ID 27), the Surinamese in the Netherlands are a tight community where it is difficult to keep things a secret: *If you talk [about diabetes] you don't get support. You get gossip. They go around and gossip about you*.

A 66-year-old African Surinamese woman (ID 28) who said *I'm not going to announce to everybody that I have diabetes *referred to 'shame': *I don't know, maybe it's shame*. One 69-year-old African Surinamese woman (ID 23) who said she would never use special facilities for people with diabetes at community gatherings such as dinner parties, gave a more elusive answer when she was asked why she wouldn't use these facilities. She said: *I always have my own food in my purse. I don't know why [I don't want to join other people with diabetes]. Maybe it's very complex*. A 54-year-old South Asian Surinamese man (ID 13) said that patients who inject insulin can carry a stigma in his community. *My wife and my closest relatives, my brothers and sisters, knew about it. Yet other people, my wife's family or friends, no, they weren't supposed to know. I was also injecting insulin, you know. In the past it was a disaster if someone knew about this*. A 58-year-old African Surinamese man (ID 10) provided perhaps a more fundamental explanation for the taboo on talking about diabetes: *They [the Surinamese] see it [diabetes] as a disgrace [for themselves] and also for their families. There are a lot of things that take place within certain families, but you never hear anything about it*. And he (ID 10) continued: *If my niece knows I have diabetes she's going to worry. And then I think, it's better that she doesn't know, since it only has drawbacks*. Indeed, not wanting to worry family members was a recurrent theme in statements about the disclosure of diabetes. A rather similar theme emerged in the statements about communication about diabetes in the workplace. Reiterating the more general perception of cultural differences between the Surinamese and native Dutch, a 54-year-old African Surinamese woman (ID 15) stated: *At work, the Dutch people with diabetes freely talk about it to others. And they also inject insulin while others are there. But Surinamese people don't do that. They think others don't have to know [that you have diabetes] and [that others] don't have to see it*. A 40-year-old South Asian Surinamese woman (ID 8) referred to fear of discrimination: *It's a chronic disease. It will never go away and I think that's the problem. During job interviews I never tell them that I have diabetes, because you might miss out on an opportunity*. A 40-year-old African Surinamese man (ID 18) referred to the absence of colleagues you can rely on: *The problem in my department is that people come and go [...] I don't have that much to do with them. So in my immediate environment, the first person I would approach about diabetes is my partner ... or my family*.

### Perception of diabetes and diabetes management

Most respondents felt that disclosure would only be necessary if the disease was serious or if certain diabetes self-management activities required this. Given the social constraints described above, it is not surprising that many of them believed they did not fall into these categories. For instance, one of these respondents, a 70-year-old African Surinamese woman, explained not talking about her diabetes by saying she did not acceptshe had it (ID 4): *I don't believe I have diabetes. It's just there for a little while*. Many respondents felt that, in their situation, talking to other people was not necessary, as they could take care of their diabetes themselves. They also felt that taking care of their diabetes was primarily an individual responsibility. A 70-year-old South Asian man (ID 27) said: *No, we don't talk about my diabetes. He [brother] knows what the doctor told me. [...] I don't talk about it. I don't see any reason. They [children] know I'm a person with diabetes, but I can do everything myself, so they don't need to worry*. The same idea was reiterated by a 54-year-old South Asian Surinamese woman (ID 7): *I don't have to tell others [...] I just have to be careful about what I eat and my life goes on. That's how I see it*.

Even though many of the Surinamese people we interviewed had been living in the Netherlands for a long time, they nevertheless perceived Surinamese cultural conventions as one of the key factors guiding the disclosure of their diabetes. There was great concern about the potentially negative social consequences of the exposure of diabetes for the individual and more importantly for the family. Within this context, it is understandable that very few respondents said they had communicated about their diabetes to people outside their very close family circles.

### Motivators (Table 3)

#### Perception of the social environment

The above-mentioned taboo on the disclosure of disease did not apply to very close family members. All respondents had mentioned their diabetes to their spouses or children at home, often immediately after diagnosis. In some cases, some other very close family members were also informed (mother, father, siblings). Respondents felt these people 'should' know, and that they could trust these close family members not to transmit this information to others. A 67-year-old African Surinamese woman (ID 12) explained " *The only people I told are my children and my sister. They know I've got it [diabetes] and they keep it to themselves*.

People who had disclosed their diabetes to the people they were living with generally appreciated the results. Some mentioned that their family members were now supporting them in carrying out certain diabetes self-care activities such as following a diet. A 40-year-old South Asian Surinamese woman (ID 8) who had told her husband and children said: *And they also became interested in diabetes. And if I overdo it a bit with what I eat my husband says, 'Watch your sugar'. So they're concerned about me*. Others mentioned they would receive understanding even if they were not always able to stick to the requirements of their therapeutic regime. A 45-year-old South Asian Surinamese woman (ID 29) stated: *If I'm feeling down or don't feel well then they [partner, children] are considerate about that*.

In answer to the question of whether there were other people they might want to talk to about their diabetes, a number of respondents mentioned 'other people who were living with diabetes', because these people would be able to understand their situation. Two respondents had found a peer with diabetes in their community with whom they felt it was safe to discuss their diabetes. For instance, a 67-year-old African Surinamese woman (ID 12) who had taken the step to approach a cousin with diabetes stated: *I often talk to my cousin now. She knows a lot because she's been terribly ill. She has diabetes herself, and arthritis [...] and we can really talk*. However, given the cultural constraints we described above, finding peers with diabetes may be difficult. A 40-year-old South Asian Surinamese woman (ID 8) explained: *If you start [talking] about diabetes people open up. Then they start talking too. They may be surprised - 'Oh, do you have it too?'. But you don't find out if, if you don't tell anybody anything yourself. But you're not going to shout [it] from the rooftops [....] *

#### Perception of diabetes

Many of the respondents who did share information about their condition with others said that their changed views on diabetes had been an important step towards disclosing their disease to others. They described that they had only been able to disclose after they had accepted their disease and after they had become aware of the complications that can arise from it. A 40-year-old African Surinamese woman (ID 17) explained: *Once I accepted it myself I was ready to just talk about it*.

#### Perception of diabetes management

Despite the cultural constraints, a few respondents mentioned that certain factors had nevertheless motivated them to share information about their diabetes with people outside their households or very close family circles. These respondents typically explained that disclosure could not be avoided if they wanted to manage their diabetes well. For example, a 49-year-old South Asian Surinamese woman (ID 30) said she would tell people at social gatherings that she had diabetes to be able to refuse certain foods. Another 66-year-old African Surinamese woman (ID 28) said she told her colleagues about her diabetes as a precautionary measure to forestall problems if she got diabetic hypoglycaemia: *I had to work with them, and if something happened they had to know [about my diabetes]*. Sometimes talking to a peer led to the realisation that disclosure is needed to get access to facilities required for their therapeutic regimen. A 54-year-old South Asian man (ID 13) who had revealed his diabetes to a peer indicated this had allowed him to improve the conditions for managing his diabetes at work: *About six years ago I met another Surinamese guy, African Surinamese, who was also giving him self insulin injection. I asked him, 'How are you injecting your insulin at work?' He said, 'I have a key for the first-aid room. There's alcohol and everything else. You can close the door and quietly give yourself a your insulin injection.' I was giving myself injections in the toilet of the locker room. Not very hygienic, and many colleagues would be walking around in the locker room. After I talked to him, I decided I should also ask my boss for the key for the first-aid room. And I got it*...

For respondents who disclosed their diabetes to people outside their very close family circles, the fear of its potential negative social consequences appeared to be balanced out by more positive experiences with the effects of disclosure. They felt that the people they chose to tell provided them with social support. A 39-year-old African Surinamese woman (ID 17) stated: *I told my employer and my colleagues and they're considerate. They understand now that sometimes I have to sit down for a while or eat some candy because of side effects from the pills*. None of them mentioned that disclosure had resulted in gossip or social disgrace.

It is interesting to note that, overall, it seemed respondents with a long history of diabetes were more prone to disclose their diabetes to people outside their immediate families.

## Discussion

In this study, we explored the perspectives of Surinamese patients with type 2 diabetes in the Netherlands on disclosing their condition to people around them. All patients had chosen to disclose their diabetes to the people they were living with and to some very close family members, but rarely to other people in their wider social networks or at work. We saw that they explained these choices within the context of cultural or community conventions with regard to the disclosure of disease. An important factor limiting the disclosure of diabetes to people outside their homes was the perception that talking about a disease in public is a taboo in the Surinamese community that may lead to gossip among community members, shame, and most importantly disgrace for the patient and their family. Fear of job discrimination was an additional factor inhibiting disclosure of diabetes at work. Indeed, only a few patients said they had taken the step to disclose their diabetes to people outside their very close family circles. The most important factor motivating this decision was linked to the acceptance of diabetes and the need to acquire information or practical facilities, or for their diabetes self-management and care.

People with a longer history of diabetes seemed to be more ready to disclose their diabetes to people outside their immediate families. This is presumably because acceptance is an important factor in disclosure, and acceptance sometimes takes time. People with a long history of diabetes were more likely to have accepted their diabetes, and this influenced disclosure. However, in contrast to other studies on the disclosure of chronic conditions[36], in the present study no sex [37] differences were found.

Our study has several limitations. First, the group we interviewed was limited to first-generation Surinamese migrants to the Netherlands with diabetes who were between 30 and 70 years old. This group consisted chiefly of people with lower to intermediate socio-economic status, and the majority were women. Consequently, our findings may not capture the perspectives of other groups of patients, for example, second-generation migrants. Future studies may want to explore the perspective on the disclosure of diabetes in these populations. Second, although the disclosure of diabetes was measured using self-reports from patients, it was not verified by seeking information from people they knew. Third, given that this study showed that concealing diabetes was common, respondents may have also withheld information from the researcher. Yet, the interviewer had a Surinamese-Dutch background, and, to stimulate openness, we strived to create an environment that was as safe as possible. Moreover, we interviewed respondents at a location of their choice, often in their own homes, and emphasised that all information would be handled confidentially.

The strength of the qualitative method chosen for this study is that it builds on the direct experiences of patients. Although the size and uniqueness of our study population do not allow for generalisation, the analysis of the data was rigorous and we interviewed a sufficient number of patients to reach data saturation, which is a criterion for sample size in qualitative studies [30]. To our knowledge, no previous research on the disclosure of diabetes has been done in Suriname. However, our results are credible in the light of studies on the disclosure of other chronic diseases. For instance, previous research has shown that fear of stigmatisation can keep individuals with HIV, mental illness, or epilepsy from disclosing their condition to people outside their immediate social environment [38,38-41]. A previous study conducted in Suriname has shown that the public disclosure of HIV by a celebrity might alter the fear of stigmatisation and motivate others to disclose their HIV to people around them, and to deal with their condition [42]. However, it is quite surprising that fear of social stigmatisation was also a reason for keeping quiet about type 2 diabetes, as it is a less visible disease and very common in Western countries.

On the basis of this study, we cannot say whether this would be any different in many other ethnic groups. However, studies among Asian Americans confirmed our findings [23,24]. Our findings are also plausible in the light of the theoretical and empirical work by Mesquita and Frijda on behaviour and emotions in collectivistic and individualistic cultural contexts [23,43]. According to these authors, in individualistic cultures, people define themselves as individuals and put their own interests ahead. In contrast, in collectivistic cultures, individuals define themselves as group members and put the interests of the group ahead of their own [44]. In Hofstede's typology for classifying cultures, Suriname was located among the collectivistic cultures, along with many Asian societies [44]. In Surinamese society, family relationships play a key role. This may explain why the Surinamese in this study were very concerned about the negative effect the disclosure of diabetes may have on the social status of their families and themselves. In addition - and in line with the propositions of Frijda and Mesquita - it is possible that the beliefs that diabetes is not serious and diabetes self-management is not difficult may be explained in part by a need to neutralise the idea that the individual, and consequently his or her family, has a problem [43]. Interestingly, we found that people who did expose their diabetes to people outside the context of their immediate families did not report any negative social consequences, but focused instead on the benefits for their diabetes.

In the public health sector, culturally sensitive community-based diabetes self-management education interventions are regarded as a potentially useful, new approach to improve diabetes management in ethnic minority populations. According to a systematic review by Netto, researchers have thus far paid attention only to a limited number of factors that may inhibit participation in such interventions, such as costs, transport, childcare, or stress associated with participation [17]. The present study suggests that people's readiness to disclose their diabetes to other people in their community may also present a barrier to participation. Based on our findings, community-based interventions involving household members and peers from their community with diabetes are probably most likely to be successful in the Surinamese target group. At the same time, these findings set the stage for future projects that aim to develop community-based diabetes self-management education for specific populations. These projects must recognise that a variety of factors may influence patients' readiness to discuss their diabetes with other people, and that these factors must be identified and addressed, especially if interventions are to include people that do not belong to the immediate families of the patients.

## Conclusions

The Surinamese patients we studied varied in their willingness to disclose their diabetes to others due to a complex mix of cultural and disease-related factors. Social conventions and expectations specific to their community could create barriers to disclosure. Community-based support interventions are currently recognised as important means for facilitating diabetes self-management [13,14,16]. Patients with type 2 diabetes may be prevented from participating in these interventions if they want to conceal their diabetes from people around them. What these findings highlight is that public health researchers and initiatives must identify and work with factors that influence the disclosure of diabetes if they are to develop community-based diabetes self-management interventions for ethnic minority populations. Attention to these factors is also needed among health care professionals, as current guidelines for diabetes care generally recommend that they should encourage their patients to seek social support for diabetes self-management activities.

## Competing interests

The authors declare that they have no competing interests.

## Authors' contributions

KS and JH initiated the study. MK conducted the interviews, checked all transcripts, and undertook data coding and initial analyses. JH was involved in checking the coding and analysis. All authors contributed to the interpretation of data and the writing of the manuscript. MK wrote the first draft of the article and all authors revised it critically for important intellectual content. All authors approved of the final version to be published.

## Pre-publication history

The pre-publication history for this paper can be accessed here:

http://www.biomedcentral.com/1471-2458/11/399/prepub
